# A threshold concentration of anti-merozoite antibodies is required for protection from clinical episodes of malaria^☆^

**DOI:** 10.1016/j.vaccine.2013.06.042

**Published:** 2013-08-20

**Authors:** Linda M. Murungi, Gathoni Kamuyu, Brett Lowe, Philip Bejon, Michael Theisen, Samson M. Kinyanjui, Kevin Marsh, Faith H.A. Osier

**Affiliations:** a KEMRI Centre for Geographic Medicine Research, Coast, P.O. Box 230-80108, Kilifi, Kenya; b Centre for Clinical Vaccinology and Tropical Medicine, Nuffield Department of Clinical Medicine, University of Oxford, Churchill Hospital, OX3 7LJ Oxford, United Kingdom; c Department of Clinical Biochemistry and Immunology, Statens Serum Institute, Copenhagen, Denmark; d Center for Medical Parasitology at Department of International Health, Immunology and Microbiology, University of Copenhagen, Copenhagen, Denmark

**Keywords:** Anti-merozoite antibodies, Protection, Clinical malaria, Protective threshold antibody concentrations, Immuno-epidemiology

## Abstract

Antibodies to selected *Plasmodium falciparum* merozoite antigens are often reported to be associated with protection from malaria in one epidemiological cohort, but not in another. Here, we sought to understand this paradox by exploring the hypothesis that a threshold concentration of antibodies is necessary for protection. We analyzed data from two independent cohorts along the Kenyan coast, one in which antibodies to AMA1, MSP-2 and MSP-3 were associated with protection from malaria (Chonyi) and another in which this association was not observed (Junju). We used a malaria reference reagent to standardize antibody measurements across both cohorts, and applied statistical methods to derive the threshold concentration of antibodies against each antigen that best correlated with a reduced risk of malaria (the protective threshold), in the Chonyi cohort. We then tested whether antibodies in Junju reached the protective threshold concentrations observed in the Chonyi cohort. Except for children under 3 years, the age-matched proportions of children achieving protective threshold concentrations of antibodies against AMA1 and MSP-2 were significantly lower in Junju compared to Chonyi (Fishers exact test, *P* < 0.01). For MSP-3, this difference was significant only among 4–5 year olds. We conclude that although antibodies are commonly detected in malaria endemic populations, they may be present in concentrations that are insufficient for protection. Our results have implications for the analysis and interpretation of similar data from immuno-epidemiological studies.

## Highlights

• We hypothesized that a threshold antibody concentration is required for protection.
• Thresholds were derived using statistical methods in a high transmission setting.
• Thresholds were applied to a low transmission setting.
• Most children had antibodies below the threshold in the low transmission setting.

## 1 Introduction

Antibodies play an important role in mediating protection against clinical malaria. Purified total IgG obtained from malaria-immune African adults was successfully used to treat children and adults hospitalized with malaria, while control sera from adults not exposed to malaria had no protective effect [1–3]. Identifying the target(s) of these “protective” antibodies continues to be a priority for malaria vaccine development. Immuno-epidemiological studies are widely used to assess the potential protective efficacy of antibodies against *Plasmodium falciparum* antigens in humans. However, such studies have often yielded inconsistent results with some studies demonstrating a protective role for antibodies to a specific antigen, while others do not [4]. One important reason for this may be the lack of a standardized approach to the reporting of antibody concentrations, and the methods used for their analysis [4,5]. While there is reasonable agreement that high levels of antibodies are better indicators of protection than sero-positivity, the definition of “high” varies considerably between studies [5–10], making it difficult to compare findings from different sites.

Here, we investigated why antibodies appeared to be protective in some settings but not in others. Specifically, we tested the hypothesis that a threshold concentration of antibody was required for protection and that in some settings, although antibodies were present, their concentrations were below the thresholds required for protection. Quantitative correlates of protection have been reported for vaccine-induced antibodies against many infectious diseases [11]. For malaria, although antibodies to several specific antigens have been shown to correlate with protection from clinical episodes of malaria [4], similar quantitative correlates have not yet been defined. In one study, the concept of an antigen-specific threshold concentration of antibodies that correlated best with protection against *P. falciparum* infection was explored [5] but not applied in subsequent studies [12,13]. In the study reported here, we develop this concept by using data from one cohort to identify “protective thresholds”, defined as the antibody concentrations against specific antigens that best correlate with protection from clinical episodes of malaria. We subsequently tested the validity of these thresholds in an independent cohort.

Our previous studies have shown that antibodies to specific merozoite antigens were associated with protection from clinical episodes of malaria in the Chonyi cohort [6,14–16]. In subsequent studies conducted in the same geographical area along the Kenyan coast, but during a period of moderate transmission, antibodies to the same panel of merozoite antigens were not associated with protection (data presented here). We used a purified IgG preparation as a reference reagent to standardize the measurement of antibody concentrations in both cohorts, and statistical methods to determine the relative IgG concentrations against each antigen that best correlated with protection in the Chonyi cohort. We show that antibody concentrations in the moderate transmission cohort were below the thresholds required for protection.

## 2 Materials and methods

### 2.1 Study populations

#### 2.1.1 Chonyi cohort

The Chonyi cohort in Kilifi, Kenya, has been extensively studied [6,14–22]. The parasite prevalence rate in children aged 2–10 years (PfPR_2–10_) [23] was 44% at the time of the study. For this report we analyzed 286 serum samples collected in October 2000 at the start of a malaria transmission season from children aged 0–10 years. These children were subsequently followed up for 6 months for clinical episodes of malaria. In this area, the age-specific criteria for defining clinical episodes of malaria are established and are as follows: for children <1 year old, a temperature of >37.5 °C plus any parasitaemia; for children >1 year old, a temperature of >37.5 °C plus a parasitaemia of >2500/μl [20]. Clinical episodes of malaria were monitored by both active and passive case detection. Trained field workers visited the participants every week whereby children with fever (axilliary temperature >37.5 °C) had a blood slide taken. Children with a positive test result were treated with antimalarial drugs. In addition, parents were advised to report to a dedicated outpatient clinic at Kilifi District Hospital if their child developed symptoms of disease at any time.

#### 2.1.2 Junju cohort

Children aged 1–6 years in a second independent group, the Junju cohort, were originally recruited in 2005 [24] and have been followed up as above, for clinical episodes of malaria [20,24]. In addition, trained field workers were available in the village to conduct passive surveillance. Children born into study households are continuously recruited into the cohort. Peak malaria transmission occurs during the rainy months of May–July and November to December. Blood samples are collected annually during a cross-sectional survey conducted at the beginning of the malaria transmission season in May. The PfPR_2–10_ in Junju was 29% at the time of sampling. Participants in the Junju cohort area live approximately 25 km away from those described above in the Chonyi cohort. We analyzed 304 serum samples collected in May 2008 from children aged 1–12 years. Data on 6 months of follow up in the subsequent malaria transmission season are presented here.

The Kenyan national scientific and ethics committees reviewed and approved the studies.

### 2.2 Recombinant *P. falciparum* merozoite antigens

All antigens are based on *P. falciparum* and include the 19 KiloDalton C-terminal fragment of merozoite surface protein (MSP)-1 of the Wellcome parasite line [25], full-length recombinant apical membrane antigen (AMA)-1 of the HB3 parasite line [26], MSP-2 of the Dd2 parasite line [27] and MSP-3 of the 3D7 allelic type [6,28]. Responses to two fragments of Glutamate-rich protein (GLURP) representing the N-terminal non-repeat region (GLURP-R0) and C-terminal repeat region (GLURP-R2) were also analyzed [29].

### 2.3 Enzyme-Linked Immunosorbent Assay (ELISA)

Serum IgG responses to individual antigens were measured using a well-established standard ELISA protocol [6,14–16]. Additionally, a purified IgG standard was incorporated into the assay and allowed for the extrapolation of relative antibody concentrations. Eleven two-fold serial dilutions of a reference Malaria Immune Globulin (MIG) reagent (Central Laboratory Blood Transfusion Service SRC, Switzerland) [30] were included for every antigen to generate a standard ELISA curve. This preparation contains 50 mg/ml of immunoglobulins (98% IgG) purified from a pool of healthy Malawian adult plasma and was originally manufactured to test its potential use as an adjunct therapy to quinine in the treatment of cerebral malaria [30]. The four-parameter logistic function was used to fit the standard curve in GraphPad Prism version 4.0 (GraphPad Software, San Diego, CA). ELISA OD values of test samples were converted into relative antibody concentrations using parameters estimated from the standard curve, assuming the purified IgG preparation contained 50 arbitrary units (AU) of antigen-specific antibodies. A pool of sera from Kilifi adults was included on every plate as a positive control. Sera from twenty Europeans served as negative controls to determine cutoff values for seropositivity, defined as mean optical density plus three standard deviations. Responses to AMA1, MSP-2, and MSP-3 were measured in both cohorts whereas those to MSP-1_19_ and GLURP were measured in the Chonyi cohort only.

### 2.4 Statistical analysis

Data analysis was performed using Stata 11 (StatCorp, TX). A modified Poisson regression model was used as previously described [6] to examine the effects of individual antibodies on the outcome, defined as a clinical episode of malaria during 6 months of follow-up. Age and reactivity to parasite schizont extract were fitted as covariates in multivariate analyses, to minimize confounding by parasite exposure. Age was fitted as a categorical variable (age bands of 0–3, 4–5, 6–7, 8–12 years), while reactivity to schizont was fitted as a continuous variable. This model was used for all the analyses described below.

We used data from all children in the Chonyi cohort to estimate the relative concentration of antibodies that best correlated with clinical protection for each antigen as follows: (i) different antibody concentrations were applied as cutoffs for high versus low responders over a range of increasing concentrations up to the maximum concentration recorded against each antigen, (ii) a modified Poisson regression model was used to calculate the risk ratio at each cutoff value, (iii) the best fitting model was selected using the log pseudolikelihood [31]. The antibody concentrations that resulted in the best fitting models were designated as “protective thresholds”. The protective thresholds were then used in two ways (i) to compare age-matched antibody levels in the Chonyi versus Junju cohorts and (ii) as cutoffs, comparing the clinical outcome of children with levels above, versus below the threshold, for each antigen in the Junju cohort.

### 2.5 Comparing analyses based on protective thresholds with those based on conventional cutoffs

Analyses using the protective threshold as a cutoff point were compared to conventional analyses where the cutoff is defined as (1) seropositivity, defined as the mean plus 3 standard deviations of negative controls and (2) high versus low antibody levels, defined previously by us as the ELISA OD level above which the risk of malaria was lower than the population's average risk of acquiring a clinical episode of malaria [6]. Importantly, analyses based on high versus low antibody levels have been defined differently in different immuno-epidemiological studies, but ultimately depend on the range of ELISA OD reactivities observed in the population under test. In contrast, the protective threshold we now propose is a fixed antibody concentration, which will not vary between study populations.

## 3 Results

### 3.1 Antibodies to merozoite antigens are not associated with protection in the Junju cohort

Children in the Junju cohort were sampled during a period of moderate malaria transmission intensity. In spite of this, antigen-specific antibodies were readily detectable in the cohort, and antibody prevalence was 67%, 31% and 26% for AMA1, MSP-2 and MSP-3, respectively. Antibody levels increased significantly with age for all antigens tested (Pearson's chi-square test for trend, *P* < 0.05).

We tested whether antibodies against these antigens were associated with a lower risk of malaria in two ways. For the first analysis we used a cutoff that compared the outcome in children that were seropositive (defined as the mean plus three standard deviations of non-malaria exposed European sera) to those that were seronegative for antibodies to these merozoite antigens. In the second analysis, we compared children that had high levels of antibodies (the ELISA OD level above which the risk of malaria was lower than the population's average risk of acquiring a clinical episode of malaria) to those that had low antibody levels to each of the antigens. In both these analyses, with the exception of MSP-3, antibodies to these antigens were not associated with protection from malaria (Table 1).

### 3.2 Identifying a threshold concentration of antibodies that best correlates with a reduced risk of malaria

Next, we tested the hypothesis that the apparent lack of protection observed in the Junju cohort could be explained by insufficient antibody concentrations. To do this, we used data from the Chonyi cohort where antibodies to these antigens had previously been studied and were known to correlate with protection from malaria [6,14–16]. Using the purified IgG as a standard for measuring relative antibody concentrations, we determined for each antigen, the relative IgG antibody concentration that best correlated with protection from malaria by selecting the model with the least log pseudolikelihood [31]. The antibody concentration thus derived was designated the “protective threshold”, and it varied for different antigens, resulting in protective efficacies ((1 − RR) × 100) of 25–56% (Table 2). Notably, this threshold concentration was higher than that defined both by seropositivity and by high antibody levels (Table 2). ELISA OD's against the R0 fragment of GLURP were highly skewed to the right and it was not possible to define high levels as described above. A protective threshold could not be identified for antibodies against MSP-1_19_.

### 3.3 Antibody concentrations are below protective thresholds in Junju cohort

**Fig. 1:**
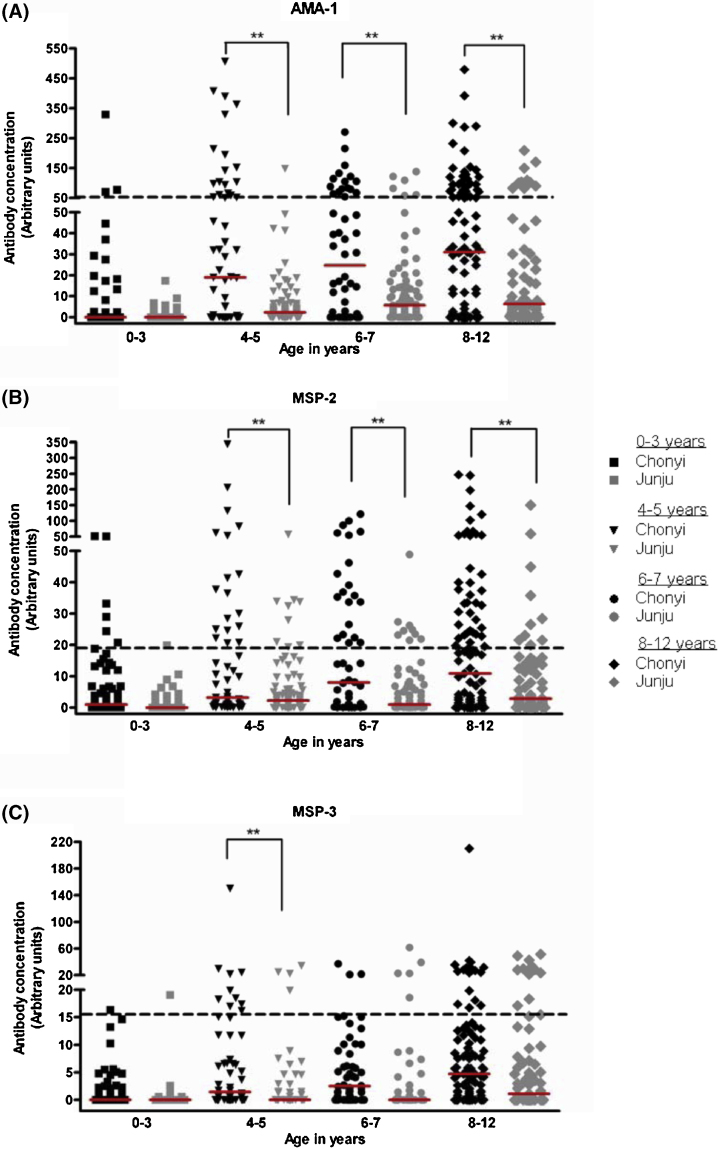
Distribution of antibody concentrations in age-matched children recruited from the Chonyi (black) and Junju (grey) cohorts against (A) AMA1, (B) MSP-2 and (C) MSP-3. Black dotted and red bold lines represent the protective threshold levels and the median antibody concentrations by age against each antigen, respectively. The proportion of individuals with antibodies above the protective threshold levels to AMA1 and MSP-2 were significantly lower in the Junju compared to Chonyi cohort across all age groups, with the exception of the youngest children. (Fisher's exact test, *P < 0.05, **P < 0.01). For MSP-3, the proportion of individuals with antibodies above the protective threshold was significantly lower in Junju compared to Chonyi only in the 4–5 year old age category. (For interpretation of the references to colour in this figure legend, the reader is referred to the web version of this article.)

We then compared age-matched antibody concentrations in both Chonyi and Junju in relation to the protective thresholds for each antigen. Median IgG levels to AMA1, MSP-2 and MSP-3 were significantly lower in the Junju cohort compared to those in the Chonyi cohort across all age groups (Mann–Whitney test, *P* < 0.01) except for the youngest children (0–3 years), who had similar median antibody levels to AMA1 in both cohorts (Fig. 1). Importantly, the proportion of age-matched children that had antibody concentrations above the protective thresholds was also significantly lower in Junju compared to Chonyi for AMA1 and MSP-2 except the youngest age-group (Fisher's exact test *P* < 0.01). The proportion of children that had protective threshold antibody concentrations against MSP-3 was significantly lower in Junju compared to Chonyi only in the 4–5 year age group (Fig. 1).

### 3.4 Protective thresholds explain apparent lack of protection in the Junju cohort

We used the protective thresholds derived in the Chonyi cohort to classify children in the Junju cohort as having antibodies above or below threshold concentrations for each antigen. We then used the modified Poisson regression model as previously described, to compare the outcome in Junju children classified in this manner. We compared these results with those obtained from analyses based on seropositive versus seronegative, and high versus low levels. For antibodies against AMA1 and MSP-3, we found that the estimates of risk decreased in a stepwise fashion, when the cutoff was applied as seropositivity, high levels, and finally as protective thresholds. For example, using these three cutoff points in multivariate analyses, antibodies against AMA1 were associated with relative risks (95% confidence intervals) of 1.09 (0.75–1.59), 0.94 (0.65–1.36) and 0.16 (0.02–1.12). For both AMA1 and MSP-3, antibody concentrations at the protective thresholds were considerably higher than those at seropositive or high levels (Table 2). For antibodies against MSP-2 the reduction in risk was modest across the three categories of analysis, and there was little difference between the protective threshold concentration and that defined by high antibody levels (Table 2). Of note, the proportion of children with antibody concentrations above any given cutoff reduced considerably as the cutoff was raised from sero-positivity, to that defined by high versus low levels, through to protective thresholds. In the Junju cohort overall, less than 10% of children had antibodies above threshold levels (5.6%, 9.5%, and 6.9% for AMA1, MSP-2 and MSP-3, respectively).

## 4 Discussion

An increasing body of evidence suggests that protection from malaria is dependent on high antibody concentrations [5–10]. Our data builds on this by using a standardized reference reagent to define the threshold concentration of antibodies for each antigen that was associated with protection against clinical episodes of malaria, using data from a high malaria transmission cohort. Application of these thresholds to an independent moderate transmission cohort provided an explanation for the lack of protection observed in the latter cohort. Antibody levels in age-matched children were significantly lower in the moderate (Junju) compared to those observed in the high (Chonyi) transmission cohort. Consequently, only a small proportion of children in Junju achieved antibody levels above the protective threshold concentrations. These data thus provide a plausible biological explanation for the observation that antibodies to individual antigens are associated with protection in some cohorts, but not others [4].

Methodological differences between immuno-epidemiological studies make it difficult to interpret apparently contradictory results where antibodies to a single antigen are associated with protection from malaria in one geographical setting, but not in another [4]. These differences range from definition of end-points, whether clinical malaria, time to infection, or malaria with high parasitaemia, for example, to duration of follow up, quality and allelic type of antigen tested, whether or not full-length or fragments of antigens were tested, right through to laboratory assays and analytical approaches, among others. In the current study, we minimized all these methodological differences and conducted the studies identically.

There was a clear difference in parasite prevalence rates in children aged 2–10 years (PfPR_2–10_) [23] in the Junju cohort (29%) compared to Chonyi (44%). Several studies have shown that parasite prevalence rates in children aged 2–10 years are reliable indicators of malaria endemicity. [23,32–34]. From these studies, areas of high transmission are defined by a PfPR_2–10_ of ≥40% whereas those of low transmission are defined by a PfPR_2–10_ of <5%. Areas of intermediate/moderate transmission which would experience an immediate reduction in parasite prevalence following large scale deployment of insecticide- treated nets have a PfPR_2–10_ of 5–40% [33,35].

Apart from differences in malaria transmission intensity, the two cohorts belong to the same ethnic group and share similar environmental factors such as cultivation practices, rainfall, wind direction, presence of streams and rivers. However, notable differences between both cohorts were the change in antimalarial drug policy from sulphadoxine–pyrimethamine to artemether lumefantrine in 2006 [36] and the distribution of free insecticide-treated bednets by the government in the same year [37] increasing coverage from 6% in 1999 [20] to more than 60% [37]. It is plausible that these factors also contributed to the decline in malaria transmission. In these circumstances, and using a malaria IgG reference serum to standardize antibody measurements across both cohorts, we were able to show antibody concentrations in the Junju cohort were significantly lower than those in Chonyi.

Immuno-epidemiological studies have traditionally classified study participants as being seropositive or seronegative for responses to specific antigens. More recently, we and others have found that classifying individuals as having high or low levels of antibodies is a highly informative indicator of protection among children [5–10]. However, the actual definition of high versus low antibody levels varies between studies, making it difficult to meaningfully compare data from different sites. We had previously derived a definition for high versus low antibody levels that was based on the range of responses observed in the cohort under study [6]. By this definition, we found that antibody levels which we would have considered to be high in Junju cohort, were nevertheless lower than those required for protection in the Chonyi cohort. Thus, an analysis based on high versus low levels in the Junju cohort would have erroneously concluded that high levels of antibodies against these merozoite antigens were not associated with protection. We therefore propose an analytical approach based on the principle of “protective thresholds” in place of (or in addition to) seropositivity, or varying definitions of high versus low antibody levels. The protective thresholds concentrations for antibodies against each antigen are fixed, and will not vary from one population to the next, allowing for efficient for comparison of data across sites. Although our estimates of risk in the Junju cohort did not always reach statistical significance, we observed a clear trend in the reduction of risk when the analysis was based on protective thresholds. In effect, this was a cutoff higher than that conventionally used for seropositivity and/or high versus low levels of antibodies. An obvious consequence of raising cutoff points is a reduction in the ability to detect significant effects, as the numbers achieving higher antibody concentrations may be relatively small, particularly in areas of low malaria endemicity. The protective threshold concentration we derived varied by antigen. For some antigens, for example AMA1 and MSP-3, this concentration was considerably higher than that defined by sero-positivity or high levels. For MSP-2, the protective threshold concentration was substantially higher than that defined by seropositivity, but was nearly equivalent to what we had previously defined as high levels (high antibody level cutoff was 18.5 AU whereas the protective threshold cutoff was 19 AU). For antibodies against GLURP, the difference between the protective threshold concentration and that defined by sero-positivity, and/or high levels (GLURP-R2) was less marked. For MSP-1_19_, we were not able to define a protective threshold by the methods presented here. These findings need to be validated in much larger cohorts, and in samples collected from different geographical settings. Efforts in this regard are underway. Additionally several studies have shown that quality of antibody responses, in particular the type of IgG subclasses are also important for protection [7,10]. We did not set out to study IgG subclasses in the current study. However, antibodies against the antigens we have analyzed here have been shown from multiple studies to comprise predominantly of the cytophilic IgG1 and IgG3 subclasses [14,16,38] even in areas of low malaria transmission intensity [39]. Thus although useful, measurement of IgG subclasses in this study would not have altered our interpretation of the data. In the present study, we measured responses to a single allelic variant of each antigen and did not ascertain whether the circulating parasite strains were bearing the haplotypes tested or whether the strains were similar in the two cohorts. Although this may be a potential limitation, our previous studies conducted in the same geographical area at different time points have found a high correlation between antibodies against different allelic versions of AMA1 [6,26], MSP-2 [6,40] and MSP-3 [6,14]. Furthermore, a longitudinal study conducted in the Gambia showed that circulating alleles remained stable over time [41].

In conclusion, our data suggests that a “protective threshold” concentration of antibodies against specific merozoite antigens of *P. falciparum* needs to be achieved for protection from clinical episodes of malaria. We propose a new approach to the analysis of such data that may add value to current analytical strategies. If validated in larger studies and in unrelated immuno-epidemiological cohorts, this analytical approach based on “protective thresholds” could be usefully extended to the testing of immunogenicity and potential protective efficacy of sub-unit vaccines currently under development for malaria.

## Figures and Tables

**Table 1:** A comparison of analyses based on antibody seropositivity, high antibody levels and protective thresholds in the Junju cohort.

	AMA-1			MSP-2			MSP-3		
	Seropos	High antibody levels	Threshold	Seropos	High antibody levels	Threshold	Seropos	High antibody levels	Threshold
	All individuals (*N* = 304)								
Prevalence	67	21	6	31	25	10	26	18	8
% (*n*/*N*)	(203/304)	(65/304)	(17/304)	(95/304)	(77/304)	(29/304)	(80/304)	(54/304)	(23/304)
IgG	42	37	6	37	35	31	30	22	13
positive^a^	(85/203)	(24/65)	(1/17)	(35/95)	(27/77)	(9/29)	(24/80)	(12/54)	(3/23)
IgG	40	42	43	43	43	42	45	45	43
negative^b^	(40/101)	(101/239)	(124/287)	(90/209)	(98/227)	(116/275)	(101/224)	(113/250)	(122/281)
Univariate	1.05	0.87	**0.13**	0.85 (0.62,1.16)	0.81	0.73	**0.66**	**0.49**	**0.33**
RR (95% CI)	(0.79,1.41)	(0.61,1.24)	**(0.02,0.91)**		(0.57,1.14)	(0.41,1.28)	**(0.46,0.95)**	**(0.29,0.82)**	**(0.11,0.95)**
Multivariate	1.09	0.94	0.16	0.79	0.78	0.74	**0.63**	**0.47**	**0.34**
RR (95% CI)	(0.75,1.59)	(0.65,1.36)	(0.02,1.12)	(0.57,1.08)	(0.55,1.12)	(0.42,1.29)	**(0.42,0.94)**	**(0.27,0.81)**	**(0.12,0.94)**

*Abbreviations*: Seropos, seropositivity; Threshold, protective threshold; ND, not determined; RR, risk ratio. The risk of developing a clinical episode of malaria during the 6-month follow-up period was compared in analyses based on antibody seropositivity, high antibody levels and protective thresholds. Data were fitted to modified Poisson regression models, adjusting for age and reactivity to parasite schizont extract in multivariate analyses [6]. Significant results at *P* < 0.05 are shown in bold.

a IgG seropositive, high antibody levels or above the protective threshold and developing malaria during follow-up.

b IgG seronegative, high antibody levels or below the protective threshold and developing malaria during follow-up.

**Table 2:** Cutoff values for antibody seropositivity, high antibody levels and protective thresholds in the Chonyi cohort.

Antigen	Seropositivity cutoff^a^ (AU)	High levels cutoff^b^ (AU)	Protective threshold^c^ (AU)	Protective efficacy^d^ (%)	(Risk ratio; 95% CI)^e^
AMA-1	0.70	43.70	55.00	25	(0.75; 0.42–1.33)
MSP-2	7.40	18.50	19.00	43	(0.57; 0.32–1.00)
MSP-3	0.39	6.50	16.00	56	(0.44; 0.16–1.17)
GLURP-R0	7.90	ND	11.00	41	(0.59; 0.27–1.28)
GLURP-R2	4.30	5.90	8.00	40	(0.60; 0.31–1.18)
MSP-1_19_	8.2	15.70	ND	ND	ND

*Abbreviations*: AU, arbitrary units; SD, standard deviation; ND, not determined. A protective threshold antibody concentration against MSP-1_19_ could not be determined. A high antibody level cutoff could not be determined for GLURP-R0 because the majority of responses against this antigen were low.

a Seropositivity cutoff is defined as the mean + 3SD response of sera from malaria naive donors for each antigen tested.

b High antibody level cutoff is defined as the antibody concentration above which the risk of malaria was lower than the population's average risk of developing malaria [6].

c Protective threshold cutoff is defined as the antibody concentration that resulted in the least log pseudolikelihood value in a modified Poisson regression model.

d Protective efficacy in analyses based on the protective threshold as a cutoff. Calculated as (1 − RR) × 100.

e Risk ratios and 95% confidence intervals applied in the calculation of protective efficacy^d^.
